# Investigation of Thermal Gel Formation of Methylcellulose in Glycols Using DSC and XRD

**DOI:** 10.3390/gels7040205

**Published:** 2021-11-09

**Authors:** Muhammad Fahad, Maqsood Ahmed Khan, Marianne Gilbert

**Affiliations:** 1 Department of Industrial & Manufacturing, NED University of Engineering & Technology, Karachi 75270, Pakistan; maqsoodahmed@neduet.edu.pk; 2 Department of Materials, Loughborough University, Loughborough LE11 3TU, UK; m.gilbertlboro@gmail.com

**Keywords:** cellulose, hydrogen bonds, DSC, XRD, gelation

## Abstract

Novel compositions of methylcellulose in ethylene, propylene and butylene glycol were investigated for their thermal gel formation. These compositions have previously been found useful for inkjet-printing-based additive manufacturing processes as support materials. Experimental techniques such as viscosity measurements between 20 °C–150 °C–20 °C, differential scanning calorimetry (DSC) and X-ray diffraction (XRD) were used and the results showed that the gel formation upon cooling is caused by polymer–polymer association. The results also show that, for methylcellulose, propylene glycol is a better solvent than ethylene glycol and butylene glycol. Since no chemical reaction is involved, these gels can be used as support materials for jetting-based additive manufacturing processes.

## 1. Introduction

Methylcellulose (MC) is a cellulose ether in which the hydroxyl (OH) groups of cellulose are replaced with OCH3 to produce methoxyl groups [1,2,3]. There are three OH groups present in each cellulose unit and in methylcellulose, none, all, or any of these can be substituted with OCH3. The process of substituting OH groups with OCH3 groups is not precisely controlled during the production of methylcellulose and thus, most of the commercially available grades of MC consist of chains (molecules) with high, low or no OCH3 substitution. The term degree of substitution (DS) is used to designate the extent of this substitution by indicating the average number of substituted OCH3 per cellulose unit in a molecule and the value of DS may vary between 3 and 0. A high DS value (~2–3) indicates highly hydrophobic behavior and the MC is soluble in organic solvents but insoluble in water. Meanwhile, a low DS value (i.e., DS~0–1.4) renders MC insoluble in nearly all solvents, including water. However, an intermediate value of DS (1.4 to 2) allows MC to be soluble in water and some organic solvents. The chains with high OCH3 groups repel water (hydrophobic) and those with high OH groups attract water (hydrophilic). This heterogeneous structure allows MC to form a gel upon heating in aqueous solutions [4,5,6,7,8]. At low temperatures, MC (DS~1.4–2) dissolves in water due to the formation of hydrogen bonds (i.e., hydration) between water and methoxy containing MC chains. When the temperature is increased, these hydrogen bonds are broken and the hydrophobicity of methoxy containing chains is increased. Breaking the water structure exposes these chains to each other and therefore, polymer–polymer interactions takes place. Depending upon the concentration of the polymer in the solution, above a certain temperature called the lower critical solution temperature or LCST, this increased hydrophobicity results in increased molecular association (i.e., polymer-polymer) interaction, and thus the solution phase separates and converts into a three-dimensional physically cross-linked gel phase. Since the gel formation does not involve making or breaking of any covalent bonds, it is considered completely reversible, and as the formed gel is cooled back, it returns to a solution phase [9,10,11,12,13]. The gel formation of MC in aqueous solution is caused due to increased polymer–polymer interactions upon heating, and the gel returns to a solution phase upon cooling [4,5,6,7,8]. The thermal gelation of methylcellulose (MC) in water has been investigated by many authors [4,5,6,7,8,9,10,11,12,13,14,15,16,17], but in non-aqueous solvents, no such studies have been performed. This research is related to the investigation of the thermal gelation of MC in glycols (i.e., without the presence of water). These non-aqueous gels could be suitable for use as support materials in jetting-based additive manufacturing processes wherein the use of aqueous gels could inhibit the polymerization of the build material. An example of such process is the jetting of caprolactam wherein the caprolactam is polymerized into nylon 6 [18,19], and the use of aqueous gels as support material could inhibit the polymerization of nylon 6. Therefore, it becomes important to use a gel without water and that can provide advantages such as easy removal from the part after build and/or reusability for multiple builds. In a previous report [20], suitability of methyl-cellulose-based materials as possible support materials for jetting-based additive manufacturing process has been demonstrated. In this paper, novel compositions of MC in ethylene glycol (EG), propylene glycol (PG) and butylene glycol (BG) have been subjected to techniques such as rheological measurements, differential scanning calorimetry (DSC) and X-ray diffraction (XRD) to investigate and explain the gel-formation mechanism of MC in glycols.

## 2. Results and Discussion

### 2.1. Rheology

**Figure 1:**
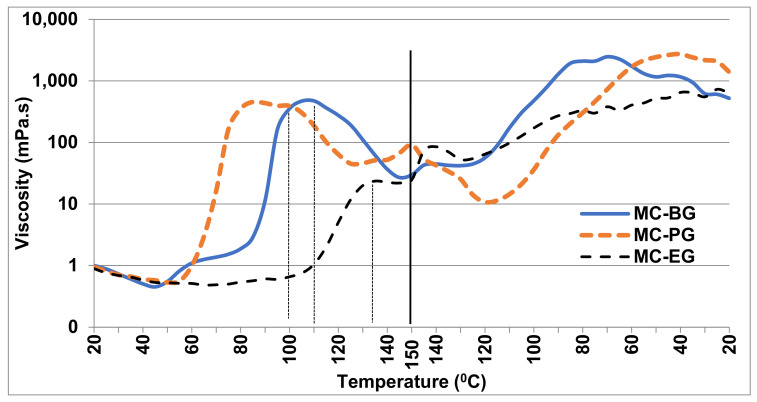
Viscosity curves for MC in ethylene glycol (EG), propylene glycol (PG) and butylene glycol (BG).

Figure 1 presents the viscosity curves of MC in all three glycols between 20 °C–150 °C–20 °C. The results show that during heating, the viscosity of each sample initially decreases but then rises to a peak value. This rise and subsequent occurrence of a peak value takes place at a noticeably different temperature for each composition. During cooling, each sample showed a gradual increase in viscosity.

### 2.2. DSC

**Figure 2:**
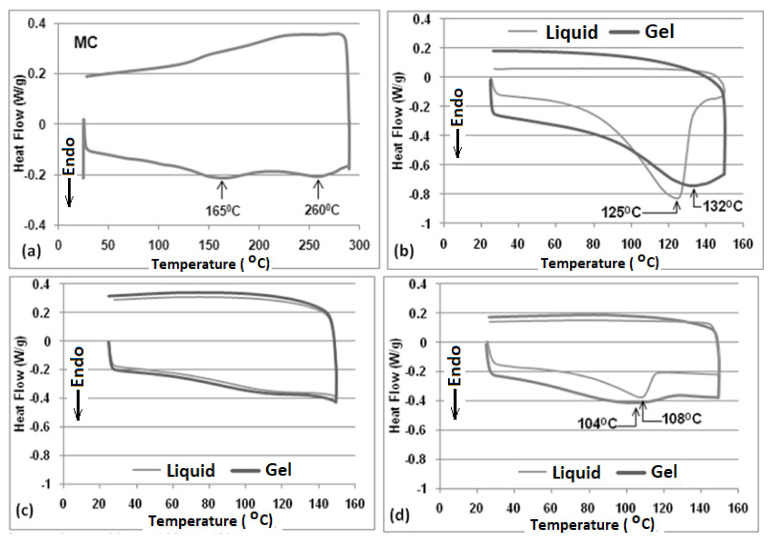
DSC curve for (**a**) MC (**b**) MC in ethylene glycol (**c**) MC in propylene glycol (**d**) MC in butylene glycol.

DSC curves for MC and MC in each glycol are shown in Figure 2. DSC of each MC in glycol sample after first heating cycle (i.e., in the gel state) was also performed, and the curves are presented in the same Figure.

DSC curve for MC (Figure 2a) shows two broad endotherms near 165 °C and 260 °C respectively. DSC curves obtained for MC in EG are presented in the Figure 2b. Endothermic peaks near 125 °C and near 132 °C were observed for the liquid and gel samples, respectively. Wide endotherms with no distinct maxima were observed for both gel and liquid MC in PG samples (Figure 2c). Similarly, Figure 2d shows an endothermic peak at 108 °C and a relatively wide endotherm with maxima at 104 °C, respectively, for the liquid and gel MC in the BG sample. During cooling, no peaks were observed for any MC in the glycol samples (both liquid and gel). Enthalpy values for all of the samples, measured via the DSC curves, are presented in the Table 1.

### 2.3. XRD

**Figure 3:**
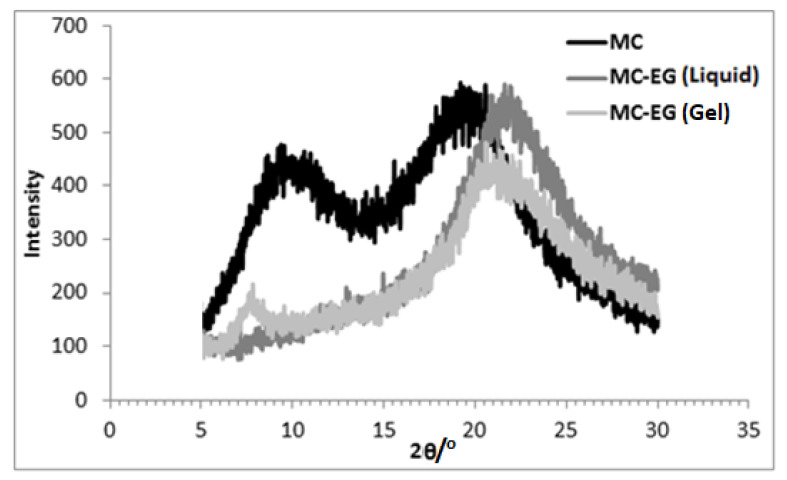
X-ray diffraction patterns for MC in ethylene glycol.

**Figure 4:**
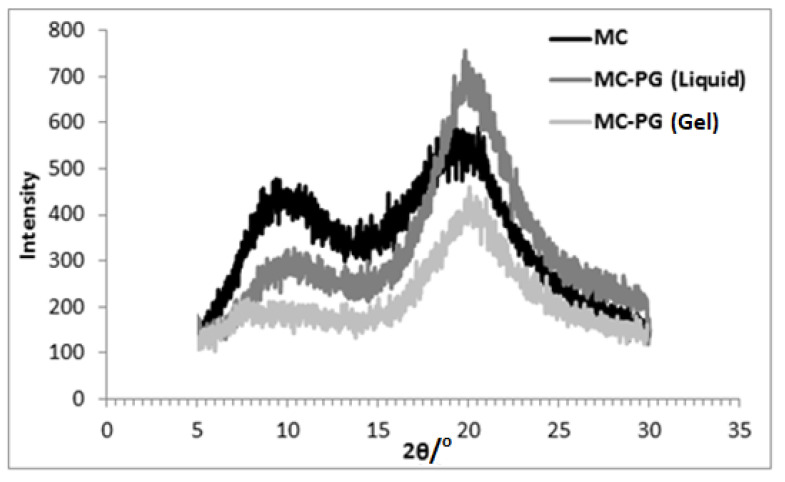
X-ray diffraction patterns for MC in propylene glycol.

**Figure 5:**
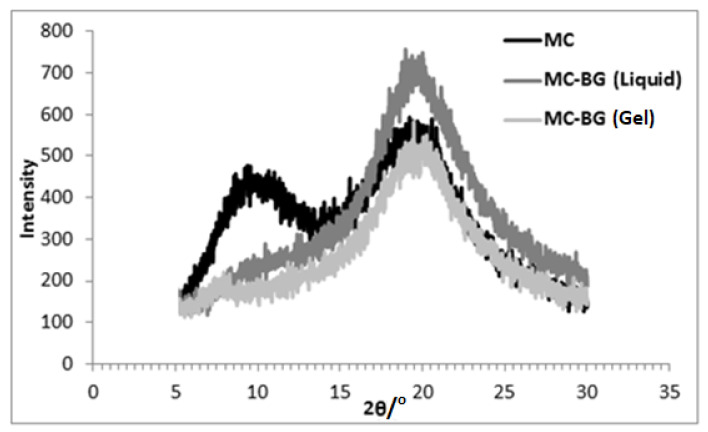
X-ray diffraction patterns for MC in butylene glycol.

X-ray diffraction results for the three different samples are presented in Figure 3, Figure 4 and Figure 5. The XRD pattern of MC powder along with the liquid and gel samples of MC in glycol is presented in each. The peaks obtained via XRD analysis and interplanar spacing (d) corresponding to each peak for different samples are listed in Table 2.

The presence of some crystalline order is evident in MC powder with two broad peaks near 2θ~9° and 19° as shown in Figure 3. For liquid MC in EG, the peak at 2θ~8° vanished, and the peak at 2θ~19° increased to 2θ~22°. For the MC in EG (gel) sample, the XRD pattern showed a low intensity and a sharp peak at 2θ~8°, and the peak intensity at 2θ~22° was reduced as indicated in Figure 3.

For MC in PG (liquid), the peak near 2θ~9° moved to 2θ~10°. An intense and sharp peak appeared near 2θ~21° (Figure 4). When the sample was heated and cooled (gel), the peak at 2θ~10° almost disappeared, whereas the peak intensity at 2θ~21° was reduced significantly.

For MC in BG (liquid), the peak near 2θ~9° vanished, and the second peak was observed at 2θ~19.5°, as shown in Figure 5. A shoulder near 2θ~7° and reduced peak intensity near 2θ~19.5° was observed for the MC in BG (gel) sample.

### 2.4. Discussion

The viscosity results (Figure 1) show that, upon heating, the viscosity slightly decreases initially and then rises steeply to a maximum value. The initial decrease in the viscosity could be due to the viscosity of the solvent itself, which decreases upon heating, but the subsequent rise could be due to the swelling of MC in the presence of the solvent. Once the MC molecules are completely swelled, the viscosity reaches a maximum value, and after that, the dissolution of MC in the glycol starts. It is clear from Figure 1 that the initial rise in the viscosity (i.e., MC swelling) and subsequent maximum value (i.e., onset of MC dissolution, marked by dotted vertical lines) takes place at a lower temperature in PG than both the EG and BG. This clearly indicates the better solvency of MC in PG, and this better solvency is attributed to the Hansen solubility parameter of PG, which is nearest to that of MC compared to the other two glycols (Table 3). Hansen solubility parameter is commonly used to determine the interactive forces responsible for compatibility between different solutes and solvents [21,22]. The total solubility parameter is divided into three main components corresponding to three different forces responsible for interaction between materials. These three forces include atomic dispersion (δ_D_), dipolar interaction (δ_P_) and hydrogen-bonding interactions (δ_H_). From Table 3, it is clear that the total solubility parameter (δ_T_) of propylene glycol is closest to MC compared with other two glycols. The rise in the viscosity during cooling represents the formation of a gel network structure, and it can be seen from the curves in Figure 1 that the rise in viscosity (i.e., gel network formation) starts at a lower temperature in PG than the other two glycols. Since PG is a better solvent, MC stays dissolved and the gel network formation takes place at a lower temperature than in EG or BG.

DSC results show two broad peaks for MC (Figure 2a). The peak at lower temperature (165 °C) could be a result of the breaking of some of the intermolecular hydrogen bonds. The peak near 260 °C represents the melting of MC, and this confirms the previous findings using hot stage microscopy, which showed the complete disappearance of the crystalline structure of MC near 268 °C [23]. The breaking of intermolecular and intramolecular hydrogen bonds caused by MC dissolution in EG (liquid) causes the heat absorbance as indicated via the endotherm in Figure 2b. No transition was observed during cooling, indicating that the formation of a network structure (i.e., gel) by MC in EG results in a less-ordered structure. For the gel state of MC in EG (i.e., after heating), a broadened endotherm was observed due to the melting of a relatively less ordered structure. For MC in PG, no identifiable peaks were observed, and for both liquid and gel samples, similar DSC curves were observed (Figure 2c). Similar results have been previously obtained for gels of MC in other solvents wherein no peaks were identified during cooling [24,25]. This is attributed to the fact that the gel-to-solution transformation is caused due to intermolecular H-bonding, whereas the solution-to-gel transformation is caused due to both intermolecular and intramolecular hydrogen bonding. Moreover, the enthalpies were much lower compared with other samples (Table 1). This could be attributed to the better dissolution of MC in PG. Because of their likeness, the solvency of PG for MC is high, and the heat flow results are relatively low compared to the other two glycols. For MC in BG, the DSC curves (Figure 2d) also suggest that, upon heating, MC structure breaks (melts) and a relatively sharp endotherm is obtained. For gel state of MC in BG, a wide endotherm was obtained upon heating which could be attributed to the melting of a less ordered/imperfect crystalline structure.

The XRD pattern (Figure 3) for MC powder shows wide diffraction peaks at 2θ~9° and 19°. Literature review has revealed similar diffraction pattern for MC [26,27,28,29,30,31,32]. Various researchers have proposed that the peak at 2θ~9° is caused by the methoxy substituted cellulose chains, whereas the peak at 2θ~19° is due to the cellulose chains with little or no substitution (high crystallinity) [10,27,28,31,33]. The XRD pattern (Figure 3) shows that, for liquid (i.e., before heating) MC in EG, the peak near 2θ~9° disappeared and the peak near 2θ~19° shifted to a higher value. Hydrogen bonding between the substituted cellulose chains, in liquid dispersion, could be a reason for these changes. In cellulose chains where OH groups are substituted with methoxy groups, the intramolecular hydrogen bonds are broken, and OH groups are available in these substituted chains to provide possible hydrogen bonding sites. Therefore, when MC is mixed with EG, hydrogen bonding takes place between EG and substituted cellulose chains present within MC. This disrupts the ordered structure present in this region resulting in the disappearance of the peak at 2θ~9°. On the other hand, the unmodified cellulose chains are forced to come closer due to this hydrogen bonding, and therefore, inter-planar distance is reduced, as indicated by the shifting of the peak at 2θ~19° to 2θ~22° (Table 2). The XRD pattern for MC in EG (gel), a sharp peak near 2θ~8° and a low intensity peak near 2θ~22° were observed. This indicates that the heating of MC in EG results in the breakage of hydrogen bonds within unsubstituted cellulose chain (intramolecular) and formation of hydrogen bonds between EG and both the ether oxygen and the OH groups within the MC molecules. This causes the disruption of MC structure indicated by reduced peak intensity (i.e., near 2θ~22°) corresponding to cellulose structure. Similarly, the peak near 2θ~8° indicates the presence of ordered chains (polymer–polymer interactions) due to interactions between methoxy-substituted MC chains. These results also confirm the findings using hot stage FTIR in a previous study [23].

For MC in PG, the diffraction pattern (Figure 4) shows the shifting of the peak near 2θ~9° towards 2θ~10°, and the peak intensity was reduced. The peak at 2θ~19° shifted to 2θ~20° with an increase in the intensity. This indicates that the mixing of MC in PG resulted in the hydrogen bonds formation between substituted (methoxy-containing) MC chains and PG. Since, the OCH_3_ groups are also present within PG molecules, the peak near 2θ~9° did not disappear completely as noticed for MC in EG sample. The hydrogen bonds between methoxy-substituted chains of MC and PG cause the unmodified cellulose chains to come closer and the interplanar distance to reduce (2θ increased to 20°). It must be noted that, for MC in PG, the peak position shift is less than that for MC in EG, which could be due to an additional carbon atom that is present in the PG molecule (increased chain length) and that results in increased interplanar distance in PG compared to in EG (Table 2). Since, among all three glycols, PG has the highest compatibility with MC, the diffraction peak at 2θ~21° for MC in PG (gel) showed considerably reduced intensity because of a highly disrupted structure compared with the other two glycols. Moreover, a sharp peak at 2θ~8° was observed for MC in EG (gel), whereas this peak broadened for MC in PG (gel), indicating less ordering between methoxy-containing chains caused by the increased solubility of MC in PG.

The diffraction pattern for MC in BG is similar to that observed for MC in EG (Figure 5). The peak at 2θ~9° turned to a low intensity, shoulder-like peak, and the peak intensity at 2θ~19° was increased. The lowering of peak intensity near 2θ~9° is attributed to the disturbance of ordering present in substituted MC chains caused by the formation of hydrogen bonds between substituted MC chains and BG. Similar to PG, OCH_3_ groups are present in the molecules of BG. But, due to an added carbon atom in BG molecule, its polarity is less than PG. Also, the difference between solubility parameters of MC and BG (Table 3) is lower than between MC and EG and higher than between MC and PG. Therefore, the peak near 2θ~9° indicated a behavior in between that of EG and PG. Hydrogen bonding between substituted cellulose chains caused the unmodified cellulose chains to come closer, resulting in increased peak intensity near 2θ~19°. The added carbon atom in the BG molecule resulted in increased interplanar distance compared with both EG and PG (Table 2). The diffraction pattern for MC in BG (gel) indicates reduced peak intensity at 2θ~19° because of a disordered structure. Similar to EG, a low-intensity peak at 2θ~8° was observed. This peak indicates that the breaking of hydrogen bonds between MC and BG during cooling exposes the methoxy groups, and polymer–polymer interactions take place, resulting in a gel structure formation. As the interaction between MC and BG is more compatible than MC in EG and less compatible than MC in PG, this peak in the BG sample is less sharp than in the EG sample and more sharp than in the PG sample.

From above discussion, it is clear that the gel formation of MC in the three glycols take place due to polymer–polymer interactions that take place during cooling. Due to its higher compatibility, the gel formation of MC in PG takes place at a lower temperature and requires less energy to form the gel, and thus, could be considered the better choice of solvent.

## 3. Conclusions

Novel compositions containing MC in three different glycols (ethylene, propylene and butylene glycols) were prepared and investigated for thermal gel formation. Experimental techniques such as viscosity measurements, DSC and XRD were performed to investigate the phenomenon of MC gel formation in glycols. The results suggested that MC dissolves upon heating in the glycol and that hydrogen bonds are formed between MC and the glycol. When these solutions were cooled, hydrogen bonds between methoxy-containing cellulose chains and the glycol were broken, causing an interaction between polymer chains (polymer–polymer association) and a gel network structure to be formed. When compared with aqueous MC gels, polymer–polymer associations take place during heating and the gel is formed at higher temperature. The results also showed that propylene glycol allows the better dissolution of MC compared with ethylene glycol and butylene glycol. Since these gels melt upon heating and reform, they could be used as support materials for inkjet-based additive manufacturing processes. These support materials can be removed easily by melting and the lack of irreversible chemical reaction involved during gel formation and subsequent melting could allow for the reusability of these materials for multiple builds.

## 4. Materials and Methods

### 4.1. Materials

Methylcellulose (viscosity of 2% aqueous solution = 12–18 mPa.s, DS~1.8, Mw~40,000), manufactured by Acros Organics, was purchased from Fisher Scientific (authorized distributors for Acros Organics products in UK). Ethylene glycol (ethanediol), propylene glycol (1,2 propanediol) and butylene glycol (1,3 butanediol) were also purchased from Fisher Scientific, and all were used as received. Table 4 lists the molecular weight and boiling point of the three solvents used in this research.

### 4.2. Sample Preparation

**Figure 6:**
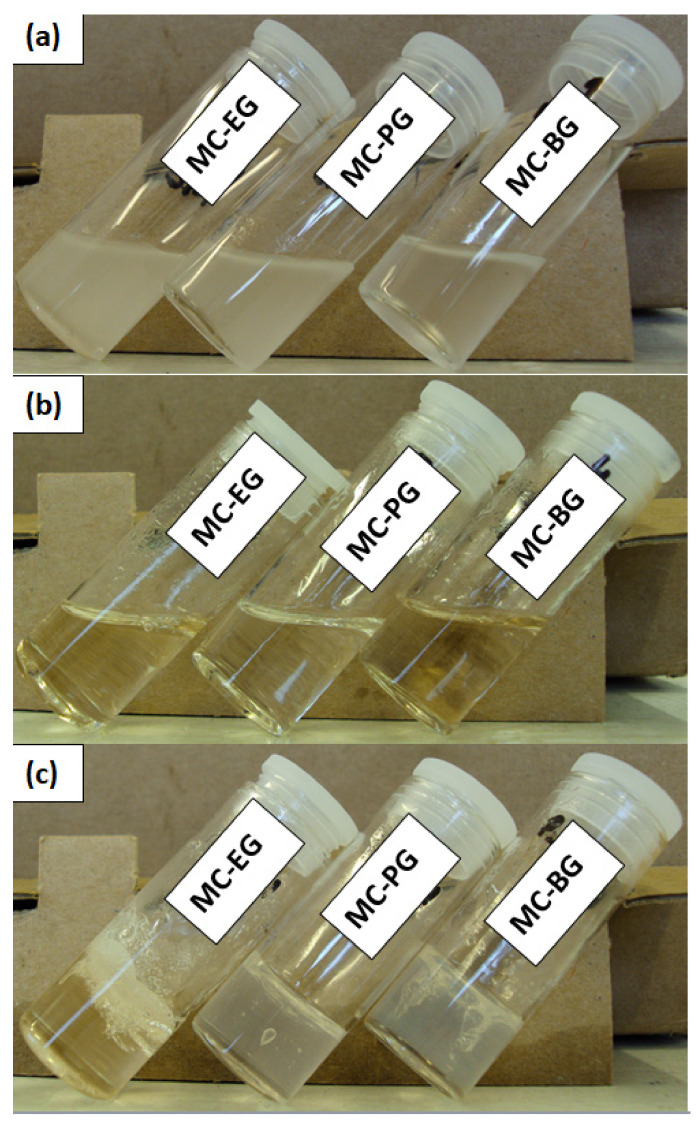
Samples of MC in EG, PG and BG at (**a**) native samples’ room temperature after mixing (liquid), (**b**) heated to 150 °C, (**c**) cooled back to room temperature (gels).

Three different samples, each containing MC (20%, *wt*/*wt*) in ethylene (EG), pro-pylene (PG) and butylene glycol (BG), were prepared. The mixing of a weighed quantity of MC in each glycol was performed via a magnetic stirrer, and each sample was allowed approximately 24 h of mixing for homogeneous dispersion. The native samples, that is, the samples immediately after mixing, are called liquid samples, and those after heating to 150 °C and cooled back to room temperature are called gels throughout this text. The samples before heating, during heating and after heating are shown in Figure 6.

### 4.3. Rheology

The viscosity of the samples was measured using Anton Paar Physica MCR 101 bench-top rheometer during heating and cooling. Each sample was heated from 20 °C–150 °C and cooled to 20 °C at 5 °C/min and a constant shear rate of 1 s^−1^ was used. A parallel plate arrangement with diameter of 25 mm and a gap of 1 mm was used for viscosity measurements.

### 4.4. Differential Scanning Calorimetry (DSC)

The samples for DSC were prepared in hermetically sealed pans. The DSC was performed on DSC Q200 equipment manufactured by TA Instruments. A nitrogen gas (N_2_) environment was used inside the heating chamber at a rate of 50 cm^3^/min. The sample of MC in each glycol was heated from 20 to 150 °C (280 °C for MC powder) and cooled back to 20 °C at a rate of 5 °C per minute (cooling and heating). The samples were held for one minute at the highest temperature to attain thermal equilibrium. Three samples of each type were tested to confirm the repeatability.

### 4.5. X-ray Diffraction (XRD)

A Bruker D8 equipment was used for XRD of the samples. The diffraction patterns for MC and the three MC in glycol samples in liquid (i.e., before heating) and gel (i.e., after heating) were obtained between 2θ = 1° to 30°. CuKα radiations (wavelength = 1.5418 Å) at 0.02°/second scan speed were used.

## Figures and Tables

**Table 1:** Enthalpy change during the heating (DSC) of samples.

Sample		Enthalpy (J/g)
20% MC–EG	Liquid	322
	Gel	61
20% MC–PG	Liquid	21
	Gel	13
20% MC–BG	Liquid	22
	Gel	22

**Table 2:** XRD peaks and corresponding interplanar (d) spacings.

Sample		2θ (Degrees)	d (Å)
Methylcellulose		9.36	9.5
		19.2	4.6
20% MC-EG	Liquid	-	-
		21.92	4.1
	Gel	7.82	11.3
		21.02	4.2
20% MC-PG	Liquid	10.0	8.8
		20.0	4.4
	Gel	7.8	11.3
		20.0	4.4
20% MC-BG	Liquid	-	-
		19.56	4.5
	Gel	7.6	11.6
		19.46	4.6

**Table 3:** Solubility parameters for MC and the three glycols [21,22].

Component	Hansen Solubility Parameter ([MPa]^1/2^)			
	δ_D_	δ_P_	δ_H_	δ_T_
MC ^1^	18.0	15.3	19.4	30.6
Ethylene glycol	17.0	11.0	26.0	32.9
Propylene glycol	16.8	9.4	23.3	30.2
Butylene glycol	16.6	10.0	21.5	28.9

^1^ Values for A4M (Dow Chemical), which has the same DS value (i.e., 1.8) as the MC used in this research.

**Table 4:** Properties of the glycols used in the research.

Component	Chemical Formula	M_W_	Boiling Point (°C)
Ethylene Glycol (EG)	C_2_H_6_O_2_	62	197
Propylene Glycol (PG)	C_3_H_8_O_2_	76	187
Butylene Glycol (BG)	C_4_H_10_O_2_	90	203
